# Microwave analogy of Förster resonance energy transfer and effect of finite antenna length

**DOI:** 10.1038/s41598-024-59824-8

**Published:** 2024-05-07

**Authors:** Kseniia Lezhennikova, Kaizad Rustomji, Pierre Jomin, Stanislav Glybovski, C. Martijn de Sterke, Jerome Wenger, Redha Abdeddaim, Stefan Enoch

**Affiliations:** 1 Aix Marseille Univ, CNRS, Centrale Marseille, Institut Fresnel, Institut Marseille Imaging, AMUTech, 13013 Marseille, France; 2 Multiwave Technologies AG, 3 Chemin du Pré Fleuri, 1228 Geneva, Switzerland; 3 School of Physics and Engineering, ITMO University, St. Petersburg, Russia; 4 Institute for Photonics and Optical Sciences (IPOS), School of Physics, University of Sydney, Sydney, NSW 2006 Australia

**Keywords:** Energy transfer, Dipole–dipole interaction, Microwave analogy to optics, Radiofrequency antenna, Förster resonance energy transfer (FRET), Near field electromagnetism, Physics, Nanophotonics and plasmonics

## Abstract

The near-field interaction between quantum emitters, governed by Förster resonance energy transfer (FRET), plays a pivotal role in nanoscale energy transfer mechanisms. However, FRET measurements in the optical regime are challenging as they require nanoscale control of the position and orientation of the emitters. To overcome these challenges, microwave measurements were proposed for enhanced spatial resolution and precise orientation control. However, unlike in optical systems for which the dipole can be taken to be infinitesimal in size, the finite size of microwave antennas can affect energy transfer measurements, especially at short distances. This highlights the necessity to consider the finite antenna length to obtain accurate results. In this study, we advance the understanding of dipole–dipole energy transfer in the microwave regime by developing an analytical model that explicitly considers finite antennas. Unlike previous works, our model calculates the mutual impedance of finite-length thin-wire dipole antennas without assuming a uniform current distribution. We validate our analytical model through experiments investigating energy transfer between antennas placed adjacent to a perfect electric conductor mirror. This allows us to provide clear guidelines for designing microwave experiments, distinguishing conditions where finite-size effects can be neglected and where they must be taken into account. Our study not only contributes to the fundamental physics of energy transfer but also opens avenues for microwave antenna impedance-based measurements to complement optical FRET experiments and quantitatively explore dipole–dipole energy transfer in a wider range of conditions.

## Introduction

The near-field interaction between two quantum emitters is largely governed by Förster resonance energy transfer (FRET), a non-radiative energy transfer mechanism occurring at nanoscale distances^1^. FRET involves energy transfer between two dipoles namely, the donor D and acceptor A separated by a subwavelength distance *R*, which can be atoms, molecules, or quantum dots, among others^2^. FRET is widely used in single-molecule biophysics^3^, light harvesting^4–7^, photovoltaics^8–10^, and molecular biosensing^11,12^. It also plays a vital key role in quantum many-body systems^13–15^, strong coupling^16–18^, and cooperative dipole–dipole interaction^19,20^. Owing to its central role, there is a growing interest in manipulating FRET by tailoring the photonic environment, similar to the control of spontaneous emission via the local density of optical states (LDOS). A wide range of results of the photonic environment on FRET have been reported, including enhancement^21–29^, no effect^30–36^, or quenching^37–39^.

An intrinsic difficulty of FRET measurements in optics is related to the fact that the energy transferred to the acceptor decays as 1/R^6^ in free-space, requiring a meticulous control over the dipole–dipole distance R in the 3–20 nm range^26,40–42^. Moreover, FRET is highly sensitive to the mutual orientation of the dipoles, which is extremely difficult to control at a molecular scale. To address these experimental challenges, we have previously proposed to conduct fundamental studies of the role of the photonic environment on dipole–dipole energy transfer in the microwave domain^23,43^. Experiments in the microwave regime with wavelengths on the centimeter scale benefit from exquisite control of the dipole–dipole distance and mutual orientations, together with the extreme sensitivity of antenna impedance measurements, far exceeding the capabilities in optics. However, the finite size of the microwave dipole antennas raises a specific challenge. While experiments in optics with quantum emitters generally can assume point dipoles, in the microwave regime, the finite size of the dipolar antennas cannot be neglected a priori, even though the antenna size remains well below the wavelength. For an accurate analysis of energy transfer in the microwave regime, it becomes necessary to account for the non-uniform electric field over the antenna length. Moreover, because extremely small antennas have a large impedance mismatch with the transmission lines and a poor radiation efficiency, microwave experiments tend to use slightly larger dipole lengths in order to achieve a sufficient signal-to-noise ratio. This adds to the necessity of a proper analysis of the finite size influence of the dipole antennas in the near-field energy transfer measurements.

Here, we develop an analytical model to describe the role of antenna length on the dipole–dipole energy transfer in the microwave regime. Our model calculates the mutual impedance *Z*_21_ of finite-length thin-wire dipole antennas without assuming a uniform current distribution. We characterize the deviation from the point dipole theory based on Green’s function formalism, and identify the conditions where longer antennas can be used, finding the right balance between power transferred and measurement accuracy. Beyond the free space configuration, we also investigate the energy transfer enhancement near a perfect electric conductor (PEC) mirror. This geometry was previously studied in the case of FRET at optical frequencies^44^. We show that for antenna lengths above λ/10 the results differ significantly from the predictions for point dipoles, highlighting the need to account for finite-size effects when interpreting antenna-based microwave energy transfer data.

## Energy transfer between finite sized antennas in free space

### Analytical model description

In optics, the molecules/atoms are much smaller than the wavelength, so FRET is typically described using a Green function formalism. The Green function ${\hat{\text{n}}}_{{\text{A}}} \cdot {\mathbf{\mathop{G}\limits^{\leftrightarrow} }}\left( {{\mathbf{r}}_{{\mathbf{D}}} ,{\mathbf{r}}_{{\mathbf{A}}} } \right) \cdot {\hat{\text{n}}}_{{\text{D}}}$ characterizes the electric field **E**(**r**_**A**_) generated by an infinitesimal, point dipole source of dipole moment |**μ**_**D**_| oriented along ${\hat{\text{n}}}_{{\text{D}}}$ located at **r**_**D**_, at the acceptor, which has an induced dipole moment |**μ**_**A**_| oriented along ${\hat{\text{n}}}_{{\text{A}}}$ located at **r**_**A**_. In these conditions, the amount of energy transferred from the donor to the acceptor is proportional to $\left| {{\hat{\text{n}}}_{{\text{A}}} \cdot {\mathbf{\mathop{G}\limits^{\leftrightarrow} }}\left( {{\mathbf{r}}_{{\mathbf{D}}} ,{\mathbf{r}}_{{\mathbf{A}}} } \right) \cdot {\hat{\text{n}}}_{{\text{D}}} } \right|^{2}$^45^. At microwave frequencies, the power transferred between two antennas is proportional to their mutual impedance *Z*_21_, which links the current I_01_ flowing in antenna 1 to the electric field, E_21_ and the induced current I_2_ in the acceptor antenna 2^46^. In other words, the mutual impedance *Z*_21_ is the ratio of the voltage generated by the induced electric field ${\text{E}}_{21}^{0} \left( {{\text{a}}_{2} } \right)$ over antenna 2 due to antenna 1, and the current *I*_01_ flowing in antenna 1, i.e.

1 $${\text{Z}}_{21} = - \frac{2}{{{\text{I}}_{02}^{*} {\text{I}}_{01} }}\mathop \smallint \limits_{{{\text{a}}_{2} = - {\text{L}}/2}}^{{{\text{L}}/2}} {\text{ E}}_{21} \left( {{\text{a}}_{2} } \right){\text{I}}_{2}^{*} \left( {{\text{a}}_{2} } \right){\text{da}}_{2} ,$$

where I_01_ and I_02_ are the amplitudes of the currents that are fed into the antennas 1 and 2 respectively and a_2_ is the coordinate over the length of antenna 2. We assume that the length L of each antenna is much larger than its wire radius, with the coordinate over the length antenna 2 denoted as ($- {\text{L}}/2{ } < {\text{a}}_{2} < {\text{L}}/2$). If the lengths *L* of the antennas are much smaller than the wavelength ($kL = 2\pi L/\lambda \ll 1)$, the electric field in Eq. (1) can be assumed to be uniform over antenna 2. The integral in Eq. (1) then simplifies to ${\text{Z}}_{21} = - \frac{{{\text{E}}_{21} {\text{L}}}}{{{\text{I}}_{01} }}$ and therefore *Z*_21_ is directly proportional to the electric field, and thus to the Green function. However, for larger antenna lengths, the constant electric field assumption cannot be used; instead, the integration in Eq. (1) must be performed using the electric field E_21_(a_2_) at each point in the domain ($- {\text{L}}/2{ } < {\text{a}}_{2} < + {\text{L}}/2$) weighted by the sinusoidal shape of the current ${\text{I}}_{2} \left( {{\text{a}}_{2} } \right) = {\text{I}}_{02} {\text{sin}}\left( {{\text{k}}\left( {{\text{L}}/2 - \left| {{\text{a}}_{2} } \right|} \right)} \right)/{\text{sin}}\left( {{\text{kL}}/2} \right)$ (valid for antennas smaller or equal to the resonant length).

We compute the impedance $Z_{21}^{0}$ for two finite antennas in free-space analytically for two configurations: the parallel configuration, when the antennas are oriented perpendicular to the line connecting their mid-points, and the aligned configuration, when the antennas are oriented parallel to this line. In free space the energy transfer in any possible orientation of the antennas can be expressed as a linear superposition of the parallel and aligned orientations.

Here we are looking for the electric field component that is parallel to the current flow in the dipole antennas. For antennas in free space

2 $${\text{E}}_{21}^{0} \left( {{\text{a}}_{2} } \right) = - {\text{i}}\frac{{{\text{ kI}}_{01} }}{{\omega \varepsilon_{0} 4\pi {\text{sin}}\left( {{\text{kL}}/2} \right)}}\left( {\frac{{{\text{exp}}\left( { - {\text{ikr}}_{1} } \right)}}{{{\text{r}}_{1} }} + \frac{{{\text{exp}}\left( { - {\text{ikr}}_{2} } \right)}}{{{\text{r}}_{2} }} - 2{\text{cos}}\left( {{\text{kL}}/2} \right)\frac{{{\text{exp}}\left( { - {\text{ikr}}_{0} } \right)}}{{{\text{r}}_{0} }}} \right).$$

where ω is the frequency of the emission, ε_0_ is the permittivity of the free space, r_0_ is the distance between the center of antenna 1 and the point a_2_ on antenna 2, r_1_ and r_2_ are the distances between the endpoints of antenna 1 and the point a_2_ on antenna 2. The distances r_1_, r_2_, and r_0_ take different values depending upon the orientation of the antennas (Table 1), where R is the distance between the central points of the dipole antennas. Substituting the induced electric field from Eq. (2) and the sinusoidal current distribution, we obtain an expression for the mutual impedance ${\text{Z}}_{21}^{0}$ for two antennas in free space

3 $${\text{Z}}_{21}^{0} = {\text{i}}\frac{{\text{ k}}}{{\omega \varepsilon_{0} 4\pi {\text{sin}}^{2} \left( {{\text{kL}}/2} \right)}}\mathop \smallint \limits_{{{\text{a}}_{2} = - {\text{L}}/2}}^{{{\text{L}}/2}} \left( {\frac{{{\text{exp}}\left( { - {\text{ikr}}_{1} } \right)}}{{{\text{r}}_{1} }} + \frac{{{\text{exp}}\left( { - {\text{ikr}}_{2} } \right)}}{{{\text{r}}_{2} }} - 2{\text{cos}}\left( {{\text{kL}}/2} \right)\frac{{{\text{exp}}\left( { - {\text{ikr}}_{0} } \right)}}{{{\text{r}}_{0} }}} \right){\text{ sin}}\left( {{\text{k}}\left( {{\text{L}}/2 - \left| {{\text{a}}_{2} } \right|} \right)} \right){\text{da}}_{2}$$

Explicit expressions for the mutual impedances in the parallel (${\text{Z}}_{{21_{{{\text{parl}}}} }}^{0}$) and aligned $({\text{Z}}_{{21_{{{\text{align}}}} }}^{0}$) configurations are given in the Supplementary Information Section S1.

**Table 1:** Distances *r*_0_, *r*_1_, and *r*_2_ used in the calculation of mutual impedances from Eq. (3) in the parallel ($Z_{{21_{{{\text{parl}}}} }}^{0}$) and aligned ($Z_{{21_{{{\text{align}}}} }}^{0}$) orientations in free-space.

	Parallel ${\text{Z}}_{{21_{{{\text{parl}}}} }}^{0}$	Aligned ${\text{Z}}_{{21_{{{\text{align}}}} }}^{0}$
r_0_	$\sqrt {{\text{R}}^{2} + {\text{a}}_{2}^{2} }$	${\text{R}} + {\text{a}}_{2}$
r_1_	$\sqrt {{\text{R}}^{2} + \left( {{\text{L}}/2 - {\text{a}}_{2} } \right)^{2} }$	${\text{R}} + {\text{a}}_{2} - {\text{L}}/2$
r_2_	$\sqrt {{\text{R}}^{2} + \left( {{\text{L}}/2 + {\text{a}}_{2} } \right)^{2} }$	${\text{R}} + {\text{a}}_{2} + {\text{L}}/2$

### Free space results

**Figure 1:**
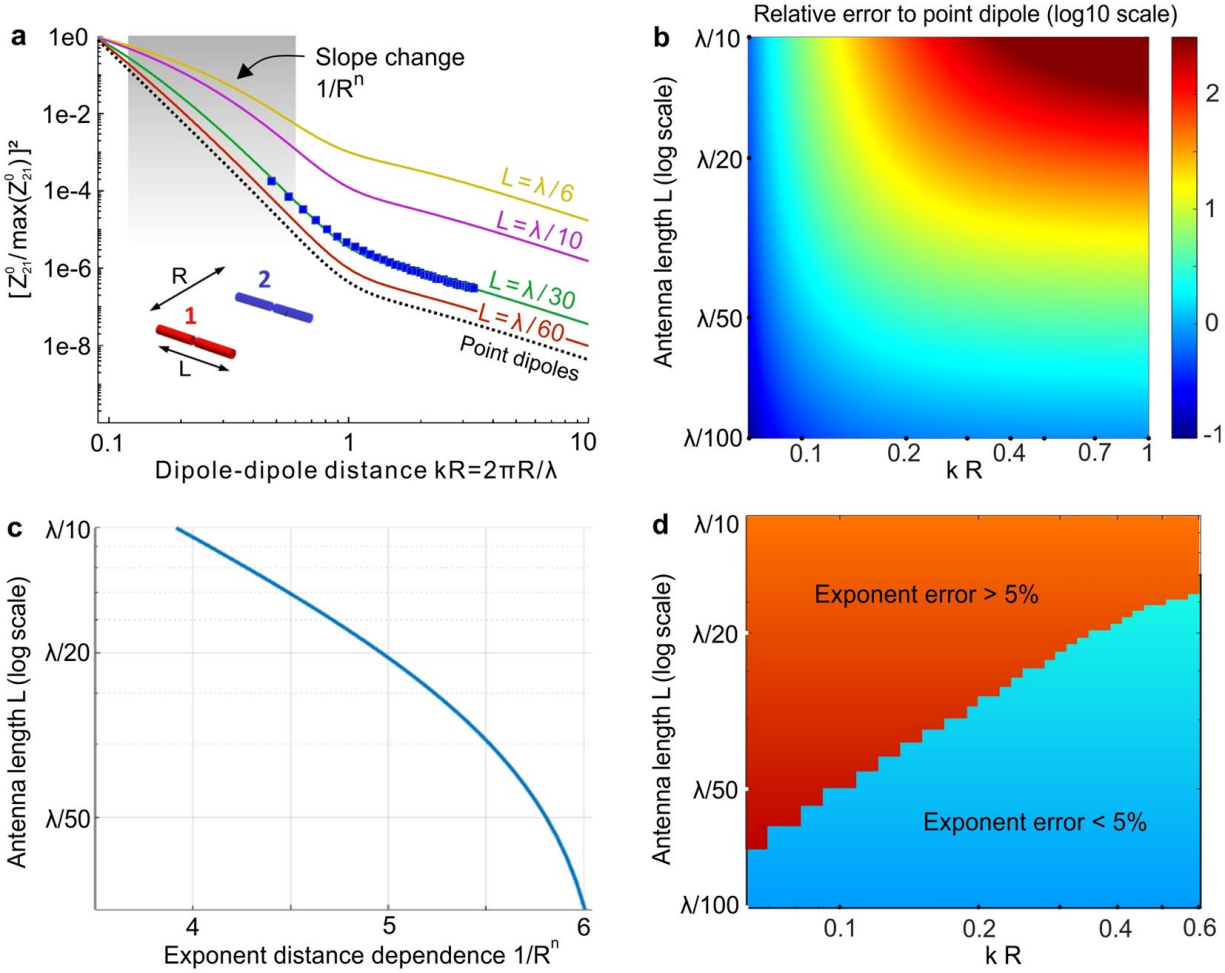
Dipole–dipole energy transfer in free space. (**a**) Calculated free space mutual impedance $\left| {{\text{Z}}_{{21_{{{\text{parl}}}} }}^{0} } \right|^{2}$ normalized by its maximum for two parallel antennas with finite lengths L. The black dotted curve corresponds to the energy transfer between ideal point dipoles from Green’s function theory, which serves as a reference. Blue markers are experimental data recorded for L = λ/30. The shaded area indicates the region considered in (**c**) to compute the exponent *n*. (**b**) Relative error (log10 scale) of the normalized mutual impedance compared to point dipoles as a function of the dipole length and mutual separation. (**c**) Distance dependence 1/R^n^ of the energy transfer averaged over the region 0.12 < kR < 0.57 (shaded area in (**a**)) as a function of the dipole length. (**d**) Bicolor map showing the {antenna length − antenna separation} regions where the exponent *n* of the energy transfer distance dependence 1/*R*^*n*^ deviates by more or less than 5% with respect to the 1/*R*^6^ dependence predicted by Green’s function theory.

We start by computing the mutual impedance normalized by its maximum $\left| {Z_{21} } \right|^{2} /{\text{max}}(\left| {{\text{Z}}_{21} } \right|{ }^{2} )$ for different antenna lengths *L* and separations *R*. Figure 1a summarizes our results for the parallel configuration, the data for the aligned configuration are given in the Supplementary Information Fig. S1. For the point dipole assumption (dotted line in Fig. 1a), the power transferred to the second antenna follows the FRET rate predictions in optics, with a 1/*R*^6^ distance dependence for *kR* < 1 which turns into 1/*R*^2^ distance dependence for *kR* > 1. For the antennas with short lengths below λ/30, the mutual impedance follows closely the point-dipole results. However, for longer dipoles (*L* > λ/30) the difference with the point dipole approximation becomes more pronounced as the finite size effects become more important. To validate our analytical model, we confirmed the results using numerical simulations with CST Microwave Studio together with experimental data (blue markers in Fig. 1a for *L* = λ /30).

Due to the finite size of the dipole antennas, the energy transfer significantly differs from the point dipole predictions. We compute in Fig. 1b the relative error respective to the point dipole predictions as a function of the antennas length and separation. As expected from Fig. 1a, the relative error grows significantly for dipole lengths above λ/30 and separations *kR* > 0.3. In contrast, dipole lengths below λ/50 remain a reasonably good approximation to the point dipole results over the entire range 0 < *kR* < 1.

A striking feature in Fig. 1a is that the evolution of the mutual impedance with the dipole–dipole separation *R* significantly deviates from the 1/*R*^6^ slope as the antenna length is increased. To account for this effect, we fit the data in Fig. 1a in the range 0.12 < *kR* < 0.57 with a 1/*R*^*n*^ dependence, and we monitor the exponent *n* (Fig. 1c). When *n* significantly differs from 6, it implies that the energy transferred deviates from the 1/*R*^6^ dependence expected for point dipoles and FRET. For antenna lengths *L* < λ/40, we find that *n* > 5.5 and is close to 6, so that finite size effects can be safely discarded. However, for lengths above *λ*/20, the energy transfer is closer to a 1/*R*^4^ dependence. Figure 1d summarizes the conditions {antenna length, dipole–dipole separation} where *n* is within 5% of the 6 exponent for point dipoles (5.7 < *n* < 6). This importantly provides guidelines to properly design the experiments in order to be able to neglect finite size effects in the interaction between dipole antennas in free space. Short antennas with *L* ~ *λ*/50 are needed to explore the entire range 0.1 < *kR* < 1. However, longer antennas with *L* ~ *λ* /20, providing a better signal-to-noise ratio, can still be used in the slightly narrower range 0.4 < *kR* < 1. Antennas with lengths exceeding λ/10 are unsuitable for investigating energy transfer in free space.

## Energy transfer near a PEC mirror

Controlling energy transfer through the electromagnetic environment is attracting increasing interest owing to many applications of FRET. Here we extend our analytical approach to investigate finite length effects on the energy transfer enhancement near a perfect electric conductor (PEC). This configuration was studied for FRET with point emitters in the optical regime, and analytical expressions are available^44^. We take advantage of the broad range of conditions accessible experimentally in the microwave domain that go well beyond earlier studies in optics (see the colormaps in the Supplementary Information Fig. S2).

**Figure 2:**
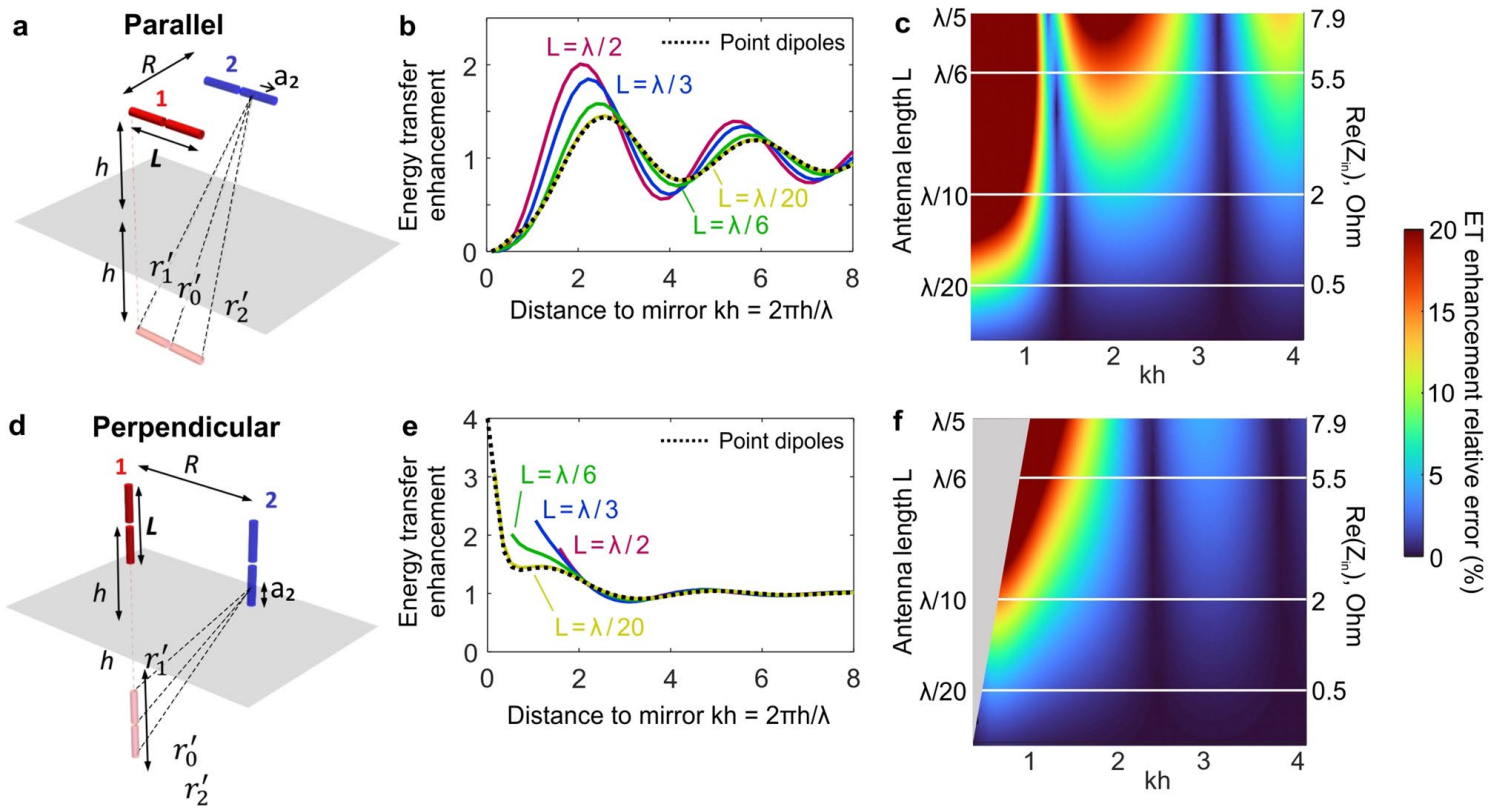
Influence of the antenna length on the energy transfer enhancement near a PEC mirror. (**a**) Setup and notations for parallel dipoles. (**b**) Energy transfer enhancement (with respect to free space) as a function of the distance to the PEC mirror for ideal point dipoles (black dotted line, Green’s function theory) and dipoles of finite lengths L (color lines, our analytical model). The separation between antennas is *kR* = 1 (*R* = 10 mm, 5 GHz frequency). (**c**) Relative error in the energy transfer enhancement of finite antennas compared to point dipoles. The right axis indicates the real part of the antenna input impedance. (**d**)–(**f**) are similar as (**a**)–(**c**), but for perpendicular dipoles. The grey area in (**f**) corresponds to the region of parameters which is physically impossible due to finite dipole lengths.

We consider the cases where both dipoles are positioned at the same distance *h* from a PEC mirror and are either parallel or perpendicular to the mirror plane (Fig. 2a,d). The case where the two dipoles are aligned is considered in the Supplementary Information Fig. S3. The electric field E_21_(a_2_) generated in the second antenna by the first one can be expressed as the sum of the field generated by antenna 1 (${\text{E}}_{21}^{0} \left( {{\text{a}}_{2} } \right)$) with current I_1_(a_1_) in the absence of the PEC mirror and its image antenna 1’ $\left( {{\text{E}}_{21}^{{\prime }} \left( {{\text{a}}_{2} } \right)} \right)$ with current ${\text{I}}_{1}^{{\prime }} \left( {{\text{a}}_{1}^{{\prime }} } \right)$,^47,48^

4 $${\text{E}}_{21}^{{{\text{PEC}}}} \left( {{\text{a}}_{2} } \right) = {\text{E}}_{21}^{0} \left( {{\text{a}}_{2} } \right) + {\text{E}}_{21}^{\prime} \left( {{\text{a}}_{2} } \right).$$

Substituting this into Eq. (4), the mutual impedance Z_21_ can be expressed as

5 $${\text{Z}}_{21}^{{{\text{PEC}}}} = \underbrace {{ - \frac{2}{{{\text{I}}_{02}^{*} {\text{I}}_{01} }}\mathop \smallint \limits_{{a_{2} = - {\text{L}}/2}}^{{{\text{L}}/2}} {\text{E}}_{21}^{0} \left( {{\text{a}}_{2} } \right){\text{ I}}_{2}^{*} \left( {a_{2} } \right){\text{dl}}_{2} }}_{{{\text{Z}}_{21}^{0} }}{ }\underbrace {{ - { }\frac{2}{{{\text{I}}_{02}^{*} {\text{I}}_{01} }}\mathop \smallint \limits_{{a_{2} = - {\text{L}}/2}}^{{{\text{L}}/2}} {\text{ E}}_{21}^{{\prime }} \left( {a_{2} } \right){\text{ I}}_{2}^{*} \left( {a_{2} } \right){\text{d}}a_{2} }}_{{{\text{Z}}_{21}^{{\prime }} }},$$

where ${\text{Z}}_{21}^{0}$ is the mutual impedance of antennas 1 and 2 in free space and ${\text{Z}}_{21}^{\prime}$ is the mutual impedance of image antenna 1’ and antenna 2. The latter can be computed using Eq. (3), with values for ${\text{r}}_{0}^{{\prime }} ,{\text{r}}_{1}^{{\prime }}$, and ${\text{r}}_{2}^{{\prime }}$ that depend on orientation as tabulated in Table 2. This results in different expressions for the mutual impedances in the parallel $\left( {{\text{Z}}_{{21_{{{\text{parl}}}} }}^{{{\text{PEC}}}} = {\text{Z}}_{{21_{{{\text{parl}}}} }}^{0} + {\text{Z}}_{{21_{{{\text{parl}}}} }}^{{\prime }} { }} \right)$, aligned (${\text{Z}}_{{21_{{{\text{align}}}} }}^{{{\text{PEC}}}} = {\text{ Z}}_{{21_{{{\text{align}}}} }}^{0} + {\text{ Z}}_{{21_{{{\text{align}}}} }}^{{\prime }}$), and perpendicular (${\text{Z}}_{{21_{{{\text{perp}}}} }}^{{{\text{PEC}}}} = {\text{ Z}}_{{21_{{{\text{perp}}}} }}^{0} + {\text{Z}}_{{21_{{{\text{perp}}}} }}^{{\prime }}$) configurations. Explicit forms for ${\text{Z}}_{{21_{{{\text{parl}}}} }}^{{\prime }} ,{\text{Z}}_{{21_{{{\text{align}}}} }}^{{\prime }} ,{\text{Z}}_{{21_{{{\text{perp}}}} }}^{{\prime }}$ can be found in the Supplementary Information. Finally, we define the energy transfer enhancement introduced by the PEC as^23,43,49^

6 $${\text{ET}}_{{\text{i}}} = \frac{{\left| {{\text{Z}}_{{21_{{\text{ i}}} }}^{{{\text{PEC}}}} } \right|^{2} }}{{\left| {{\text{Z}}_{{21_{{\text{ i}}} }}^{0} } \right|^{2} }},{ }$$

where *i* denotes the configuration: parallel, perpendicular or aligned.

**Table 2:** Distances $r_{0}^{{\prime }} ,r_{1}^{{\prime }}$, and $r_{2}^{\prime}$ used in the calculation of mutual impedance between image antenna 1’ and antenna 2 in the parallel (Fig. 2a), aligned (Fig. S3), and perpendicular (Fig. 2d) configurations.

	Parallel ${\text{Z}}_{{21_{{{\text{parl}}}} }}^{\prime}$	Aligned ${\text{Z}}_{{21_{{{\text{align}}}} }}^{\prime}$	Perpendicular ${\text{Z}}_{{21_{{{\text{perp}}}} }}^{\prime}$
${\text{r}^{\prime}}_{0}$	$\sqrt {{\text{R}}^{2} + 4{\text{h}}^{2} + a_{2}^{2} }$	$\sqrt {4{\text{h}}^{2} + \left( {{\text{R}} + {\text{a}}_{2} } \right)^{2} }$	$\sqrt {{\text{R}}^{2} + \left( {2{\text{h}} + {\text{a}}_{2} } \right)^{2} }$
${\text{r}^{\prime}}_{1}$	$\sqrt {{\text{R}}^{2} + 4{\text{h}}^{2} + \left( {{\text{L}}/2 - a_{2} } \right)^{2} }$	$\sqrt {4{\text{h}}^{2} + \left( {{\text{R}} - {\text{L}}/2 + a_{2} } \right)^{2} }$	$\sqrt {{\text{R}}^{2} + \left( {2{\text{h}} - {\text{L}}/2 + a_{2} } \right)^{2} }$
${\text{r}^{\prime}}_{2}$	$\sqrt {{\text{R}}^{2} + 4{\text{h}}^{2} + \left( {{\text{L}}/2 + a_{2} } \right)^{2} }$	$\sqrt {4{\text{h}}^{2} + \left( {{\text{R}} + {\text{L}}/2 + {\text{a}}_{2} } \right)^{2} }$	$\sqrt {{\text{R}}^{2} + \left( {2{\text{h}} + {\text{L}}/2 + {\text{a}}_{2} } \right)^{2} }$

Figure 2b and e show the energy transfer enhancement as the dipoles approach the mirror, for different antenna lengths (L = λ/20, λ/6, λ/3, and λ/2). These results from our analytical model are compared to the predictions for perfect point dipoles based on the Green’s function. The length L = λ/20 nicely interpolates the results for point dipoles, indicating that finite length effects can be discarded in this case. It also appears that the energy transfer enhancement computed in Fig. 2b,e is less sensitive to the antenna finite length than the absolute value of the mutual impedance Z_21_ in Fig. 1a. This is because the energy transfer enhancement is a ratio and therefore some deviations introduced by the finite size of the dipoles affect the results in free space and with the PEC mirror in similar ways, so that the net influence cancels out. However, this argument no longer holds when the antenna length exceeds λ/6 for which significant deviations from the point dipole predictions is observed.

To quantify deviations from point dipole theory due to finite antenna size, we compute in Fig. 2c, f the relative error of the energy transfer enhancement with respect to point dipoles as a function of the antenna length L and the distance to the mirror h. The grey areas in the colormaps correspond to the positions that are not physically accessible due to the antenna finite length. We also show the corresponding real part of the antenna input impedance in the vacuum for each length. This quantity, the radiation resistance^46^, determines the radiation efficiency (radiated power at a given fed current) of the antennas. In experiments the optimum antenna length is a balance between improving the signal-to-noise ratio by making the antenna longer, and better approximating a point dipole, by making the antenna shorter. Panels c and f of Fig. 2 provide quantitative guidelines so as to choose the antenna length.

For antenna lengths L < λ/20, the relative error remains below 10% for all the configurations and distances to mirror, so that finite size effects can be safely neglected. However, increasing the antenna length up to λ/2 can have a significant impact on the energy transfer enhancement with an error greater than 20%. Nevertheless, longer antennas with L ~ λ/10 providing a better signal-to-noise ratio can still be used, provided that the height range is limited to kh > 1. Antennas with lengths above λ/5 are unsuited for the study of the PEC influence on the energy transfer enhancement.

## Experimental validation

**Figure 3:**
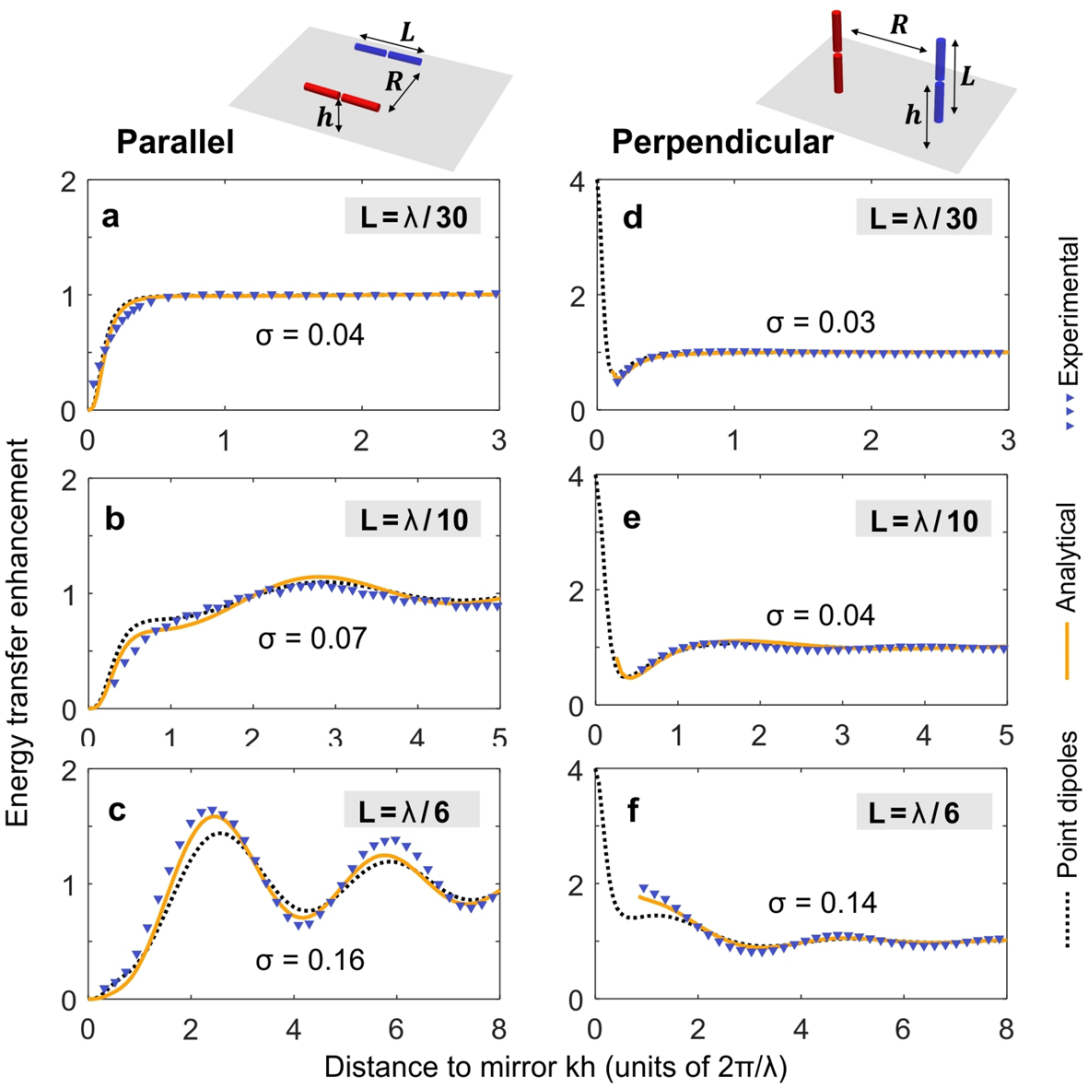
Experimental validation of the antenna length influence on the energy transfer enhancement near a PEC mirror. (**a**)–(**c**) are for parallel dipoles and (**d**)–(**f**) for perpendicular dipoles, as pictured in the top schematics. Blue triangles are experimental results, orange curves are the result of our analytical model and the dotted black curves are predictions from Green’s function theory. The antenna length normalized by the wavelength L/λ varies for each line, as indicated on the graph, and the separation is kept constant to the antenna length R = L. The root mean square errors *σ* between the experimental results and the Green’s theory predictions are shown in each graph.

Figure 3 shows experimental results directly validating our analytical model taking into account the finite length of the antenna in the case of the energy transfer enhancement near a PEC mirror. The antennas were fabricated from a coaxial cable with overall length of 4 cm. The antenna length in all experiments was L = 10 mm. We show results for frequencies of 1 GHz (Fig. 3a,d), 3 GHz (Fig. 3b,e), and 5 GHz (Fig. 3c,f). The antenna separation was kept constant at R = 10 mm, which corresponds to λ/30, λ/10 and λ/6 at 1 GHz, 3 GHz, and 5 GHz, respectively. To ensure structural rigidity, we secured the two antennas on a foam spacer with a dielectric permittivity ε_r_≈1.08. The mirror was a 1 m × 1 m square copper sheet. The antennas were connected to an Anritsu model MS2036C vector network analyzer (VNA) via 1-m-long coaxial cables. Prior to data collection, the VNA was calibrated from 1 to 5 GHz, ensuring accurate data acquisition. The choice of a 10 mm antenna length aimed to balance deviations from a point dipole while maintaining sufficient signal. We nevertheless needed to measure extremely low signals on the order of − 50 dB in the parallel configuration. An Intermediate Frequency bandwidth (IFBW) of the VNA was 500 Hz it gave us higher resolution for measurements and a dynamic range up to 120 dB for accurate measurements. In the perpendicular case, the signal power was even lower, necessitating the use of a specialized electric field probe (MVG SAR PROBE SN 17/21 EP353). Measured voltage was up to 3 × 10^−8^ V. A technical error for the electric field probe is around 1 × 10^−9^ V, which is smaller than the data points on the graphs and does not affect the calculations of the FRET rate enhancement.

To vary the distance h between the antenna and the mirror, we used a mechanical stage with a step size Δh = 1 mm, enabling us to achieve high spatial resolutions ranging from Δh = λ/60 at 5 GHz to Δh = λ/300 at 1 GHz. We used the same probe for the free space measurements shown in Fig. 1 and Figure S1. The setup was identical to the measurements near PEC mirror, just with the mirror removed.

Figure 3 presents the experimental data by triangles, together with the results of our analytical model (orange curve) which accounts for the finite antenna length, and the predictions using the point-dipole Green’s function (dotted curves). While Fig. 3 shows the results for the parallel and perpendicular case, the aligned configuration is displayed in Fig. S3. By keeping the separation R = L constant, the different curves in Fig. 3 enable us to probe different kR values, leading to different conditions. kR = 0.2 in Fig. 3a,d, kR = 0.6 in Fig. 3b,e, and kR = 1 in Fig. 3c,f. For all the different configurations, we find a remarkable agreement between the experimental data and our analytical model. This experimentally validates our approach. It also shows that finite length effects must be taken into account to properly address the energy transfer enhancement, especially for antennas with lengths above λ/10. As expected, the root mean square errors σ between the experimental data and the Green’s function theory increase with the antenna length L.

## Conclusions

Energy transfer measurements using microwaves offer more precise control over dipole positions and orientations compared to optical experiments. Earlier works assumed that the microwave antennas were small enough so that the field could be considered uniform across them, allowing a point dipole Green’s function formalism to be used for interpreting the data. However, this approach is impractical because short antennas lead inevitably to weak signals. The analytical model developed in this study explicitly accounts for the effect of longer antenna sizes on energy transfer measurements, by calculating the mutual impedance between finite-sized antennas. We investigated energy transfer between antennas placed next to a PEC mirror, validating our model with experimental measurements.

Importantly, we provide for the first time clear guidelines to properly design the microwave experiments, indicating the conditions where the finite size effects can be neglected and where they need to be taken into account. For studying energy transfer in free space, antennas with lengths around λ/50 provide the best accuracy for 0.1 < kR < 1, yet longer antennas with L ~ λ/20 can still be used for 0.4 < kR < 1 where they provide a better signal-to-noise ratio. Antennas with lengths larger than λ/10 should not be considered for the study of energy transfer in free space.

For the enhancement of energy transfer in the presence of a mirror, the conditions are somewhat relaxed since part of the finite size effects tend to cancel out in the computation of the enhancement ratio. Antenna lengths below λ/20 provide accurate measurements of the energy transfer enhancement for all the configurations and longer antennas with L ~ λ/10 can be used for kh > 1. However, antennas with lengths above λ/5 should not be used in this case. We note that experiments using larger antennas can provide valuable insights into various aspects of antenna behavior beyond FRET interactions. These studies contribute to our understanding radiation patterns, impedance matching, and other relevant phenomena relevant to antenna design and performance. Additionally, they offer opportunities to explore energy transfer mechanisms in regimes where FRET theories cannot be directly tested.

Overall, this study advances the potential for microwave antenna impedance-based measurements to complement optical FRET experiments. As optical phenomena like lossy surface plasmons^50,51^, molecular aggregates^52^ and metasurfaces^49^ are now accessible in the microwave regime, we anticipate that microwave antennas can be used to study a variety of problems in near-field optics.

## Supporting information

Full analytical forms of the mutual impedance in free-space, Energy transfer in free space between aligned dipoles, Energy transfer enhancements near a PEC mirror, Energy transfer between aligned dipoles in presence of a PEC mirror.

## Supplementary Information

Supplementary Information.

## Data Availability

The datasets used and/or analysed during the current study available from the corresponding author on reasonable request.
