# Supplementary material for: Microwave analogy of Förster resonance energy transfer and effect of finite antenna length
Source: Sci Rep. 2024 May 7;14:10485. doi: 10.1038/s41598-024-59824-8 (PMC11551205; doi:10.1038/s41598-024-59824-8)
Supplement: Supplementary file 1 — Supplementary Information. [file 41598_2024_59824_MOESM1_ESM.docx]

**Supplementary Information for**

**Microwave analogy of Förster resonance energy transfer and effect of finite antenna length**

Kseniia Lezhennikova ^1,2*^, Kaizad Rustomji^1^, Pierre Jomin^1^, Stanislav Glybovski^3^, C. Martijn de Sterke^4^, Jerome Wenger^1^, Redha Abdeddaim^1^, Stefan Enoch^1^

^1^ Aix Marseille Univ, CNRS, Centrale Marseille, Institut Fresnel, Institut Marseille Imaging, AMUTech, 13013 Marseille, France

^2^ Multiwave Technologies AG, 3 Chemin du Pré Fleuri 1228, Geneva, Switzerland

^3^ School of Physics and Engineering, ITMO University, St. Petersburg, Russia

^4^ Institute for Photonics and Optical Sciences (IPOS), School of Physics, University of Sydney,

Sydney NSW 2006, Australia

** Corresponding author:* [kseniia.lezhennikova@fresnel.fr](mailto:kseniia.lezhennikova@fresnel.fr)

**Contents:**

S1. Full analytical forms of the mutual impedance in free-space

S2. Energy transfer in free space between aligned dipoles

S3. FRET rate enhancement between point dipoles near a PEC mirror

S4. Energy transfer between aligned dipoles in presence of a PEC mirrror

# **S1. Full analytical forms of the mutual impedance in free-space**

Here we present full analytical forms of the mutual impedances $Z_{21}^{0}$ for free-space by explicitly computing the integrals in Eq. (3) and near PEC mirror from Eq. (5). For the parallel orientation in free‑space we substitute, $r_{0}=\sqrt{R^{2}+{a_{2}}^{2}}$, $r_{1}=\sqrt{R^{2}+\left( L/2-a_{2} \right)^{2}}$, and $r_{2}=\sqrt{R^{2}+\left( a_{2}+L/2 \right)^{2}}$ (Table I) into Eq. (3) to obtain

|  | $Z_{{21}_{\mathrm{parl}}}^{0}=2i\frac{k}{\omega\varepsilon_{0}4\pi\mathrm{si}n^{2}(kL/2)}[[1+2cos(kL/2) exp(-ikL/2)][Ci(u_{0})-Ci(u_{2})-iSi(u_{0})+iSi(u_{2})]+$ | |  |
| --- | --- | --- | --- |
|  |  | $+[1+2cos(kL/2)exp(ikL/2)][Ci(u_{0})-Ci(u_{2}^{'})-iSi(u_{0})+iSi(u_{2}^{'})]$ | (A1) |
|  |  | $+exp(ikL)[Ci(u_{1}^{'})-Ci(u_{2}^{'})-iSi(u_{1}^{'})+iSi(u_{2}^{'})]$ |  |
|  |  | $+exp(-ikL)[Ci(u_{1})-Ci(u_{2})-iSi(u_{1})+iSi(u_{2})]].$ |  |

Where $\mathrm{Si}\left( x \right)$ and $Ci(x)$ are the Sine and Cosine integrals: $\mathrm{Si}\left( x \right)=\int_{0}^{x} \sin\left( t \right)/t dt$ and $\mathrm{Ci}\left( x \right)=-\int_{0}^{x} \cos\left( t \right)/t dt$,

$u_{0}=kR$ ,

$u_{1}=k\left( \sqrt{R^{2}+L^{2}}-L \right)$, $u_{1}^{'}=k\left( \sqrt{R^{2}+L^{2}}+L \right)$,

$u_{2}=k\left( \sqrt{R^{2}+L^{2}}-L \right)$, and$u_{2}^{'}=k(\sqrt{R^{2}+{(L/2)}^{2}}+L/2$.

For the aligned orientation in free‑space we first divide the integration domain in Eq. (3) into two parts

|  | $\begin{matrix} Z_{{21}_{\mathrm{align}}}^{0}=\frac{i k}{\omega\varepsilon_{0}4\pi\mathrm{si}n^{2}(kL/2)}[\int_{a_{2}=0}^{L/2} \left( \frac{exp(-ikr_{1})}{r_{1}}+\frac{exp(-ikr_{2})}{r_{2}}-2cos(kL/2)\frac{exp(-ikr_{0})}{r_{0}} \right) \\ sin(k(L/2-l_{2}))da_{2}+\int_{a_{2}=-L/2}^{0} \left( \frac{exp(-ikr_{1})}{r_{1}}+\frac{exp(-ikr_{2})}{r_{2}}-2cos(kL/2)\frac{exp(-ikr_{0})}{r_{0}} \right), \\ sin(k(L/2+l_{2}))d{a_{2}}_{2}], \end{matrix}$ |  |
| --- | --- | --- |

then substitute, $r_{0}=R+a_{2}$, $r_{1}=R+a_{2}-L/2$, and $r_{2}=R+a_{2}+L/2$ (Table I) into Eq. (3) to obtain the solution

|  | $Z_{{21}_{\mathrm{align}}}^{0}$ | $=\frac{i k}{2\omega\varepsilon_{0}4\pi\mathrm{si}n^{2}\left( \mathrm{kL}/2 \right)}\left[ -ln\left( \frac{R-L/2}{R-L} \right) exp(-iu_{1}) \right.$ |  |
| --- | --- | --- | --- |
|  | | $+[Ci(2u_{2})-Ci(2u_{1})-iSi(2u_{2})-iSi(2u_{1})]exp(iu_{1})$ |  |
|  | | $+ln\left( \frac{R+L}{R+L/2} \right)\exp\left( -iu_{1}^{'} \right)-\left[ \mathrm{Ci}\left( 2u_{1}^{'} \right)-Ci\left( 2u_{2}^{'} \right)-iSi\left( 2u_{1}^{'} \right)-iSi\left( 2u_{2}^{'} \right) \right]\exp\left( iu_{1}^{'} \right)$ |  |
|  | | $+ln\left( \frac{R}{R-L/2} \right)\left[ \exp\left( -iu_{0} \right)+2cos\left( \mathrm{kL}/2 \right)\exp\left( -iu_{2} \right) \right]$ | (A2) |
|  | | $-\left[ \mathrm{Ci}\left( 2u_{0} \right)-Ci\left( 2u_{2} \right)-iSi\left( 2u_{0} \right)-iSi\left( 2u_{2} \right) \right]\left[ \exp\left( iu_{0} \right)+2cos\left( \mathrm{kL}/2 \right)\exp\left( iu_{2} \right) \right]$ |  |
|  | | $-ln\left( \frac{R+L/2}{R} \right)\left[ \exp\left( -iu_{0} \right)+2cos\left( \mathrm{kL}/2 \right)\exp\left( -iu_{2}^{'} \right) \right]$ |  |
|  | | $\left. \begin{matrix} \\ +[Ci(2u_{2}^{'})-Ci(2u_{0})-iSi(2u_{0})-iSi(2u_{2})][exp(iu_{0})+2cos(kL/2)exp(iu_{2}^{'})] \\ \end{matrix} \right].$ |  |

Where $u_{0}=kR$,

$u_{1}=k(R-L)$, $u_{1}^{'}=k(R+L)$,

$u_{2}=k(R-L/2)$, and $u_{2}^{'}=k(R+L/2)$.

To obtain the mutual impedances $Z_{21}^{PEC}$ with the mirror, in the parallel orientation we substitute
$r_{0}^{'}=\sqrt{R^{2}+4h^{2}+{a_{2}}^{2}}$, $r_{1}^{'}=\sqrt{R^{2}+4h^{2}+\left( L/2-a_{2} \right)^{2}}$, and $r_{2}^{'}=\sqrt{R^{2}+4h^{2}+\left( L/2+a_{2} \right)^{2}}$ (Table II) into the second term on the right hand side of Eq. (5) to obtain

|  | $-Z_{{21}_{\mathrm{parl}}^{'}}$ | $=\frac{k}{2\omega\varepsilon_{0}4\pi\cdot\mathrm{si}n^{2}(kL/2)}[[Ci(u_{0})-Ci(u_{2})-iSi(u_{0})+iSi(u_{2})](1 +2cos(kL/2) exp(-ik(L/2)))$ |  |
| --- | --- | --- | --- |
|  | | $+[Ci(u_{0})-Ci(u_{2}^{'})-iSi(u_{0})+iSi(u_{2}^{'})](1+2cos(kL/2)exp(ik(L/2)))$ | (A3) |
|  | | $+[Ci(u_{0})-Ci(u_{2}^{'})-iSi(u_{0})+iSi(u_{2}^{'})](1+2cos(kL/2)exp(ik(L/2)))$ |  |
|  | | $+[Ci(u_{1}^{'})-Ci(u_{2}^{'})-iSi(u_{1}^{'})+iSi(u_{2}^{'})]exp(ikL)$ |  |
|  | | $+[Ci(u_{1})-Ci(u_{2})-iSi(u_{1})+iSi(u_{2})]exp(-ikL)]$ |  |

Where $u_{0}=k\sqrt{R^{2}+(2h)^{2}},$

$u_{1}=k(\sqrt{R^{2}+(2h)^{2}+L^{2}}-L)$, $u_{1}^{'}=k(\sqrt{R^{2}+(2h)^{2}+L^{2}}+L)$,

$u_{2}=k(\sqrt{R^{2}+(2h)^{2}+(L/2)^{2}}-L/2),$ and $u_{2}^{'}=k(\sqrt{R^{2}+(2h)^{2}+(L/2)^{2}}+L/2).$

For perpendicular and aligned cases $Z_{21}^{PEC}$ with the mirror, in the perpendicular and aligned orientations we substitute the associated forms of $r_{0}^{'}$, $r_{1}^{'}$, and $r_{2}^{'}$ from Table II into the second term on the right hand side of Eq. (5) and obtain similar forms for $Z_{{21}_{\mathrm{perp}}^{'}}$ and $Z_{{21}_{\mathrm{align}}^{'}}$albeit with difference of a negative sign, which represents the out-of-phase current flow in image antenna 1’ when antenna is in aligned orientation with respect to the PEC mirror

| $Z_{{21}_{\mathrm{perp}}^{'}}=-Z_{{21}_{\mathrm{align}}^{'}}$ | $=\frac{k}{2\omega\varepsilon_{0}4\pi\cdot\mathrm{si}n^{2}\left( \frac{\mathrm{kL}}{2} \right)}\left[ \begin{matrix} \\ [Ci(u_{0})-Ci(u_{3})-iSi(u_{0})+iSi(u_{3})] \\ \end{matrix} \right.$ |  |
| --- | --- | --- |
|  | $\times(exp(ik(2h))+2cos(kL/2)exp(ik(2h-L/2)))$ |  |
|  | +$\left[ \mathrm{Ci}\left( u_{0}^{'} \right)-Ci\left( u_{3}^{'} \right)-iSi\left( u_{0}^{'} \right)+iSi\left( u_{3}^{'} \right) \right]\left( \exp\left( -ik\left( 2h \right) \right)+2\cos\left( \frac{\mathrm{kL}}{2} \right)\exp\left( -ik\left( 2h-\frac{L}{2} \right) \right) \right)$ |  |
|  | $+\left[ \mathrm{Ci}\left( u_{0}^{'} \right)-Ci\left( u_{3}^{'} \right)-iSi\left( u_{0}^{'} \right)+iSi\left( u_{3}^{'} \right) \right]\left( \exp\left( -ik\left( 2h \right) \right)+2\cos\left( \frac{\mathrm{kL}}{2} \right)\exp\left( -ik\left( 2h-\frac{L}{2} \right) \right) \right)$ |  |
|  | $+\left[ \mathrm{Ci}\left( u_{0} \right)-Ci\left( u_{4} \right)-iSi\left( u_{0} \right)-iSi\left( u_{4} \right) \right]\left( \exp\left( \mathrm{ik}\left( 2h \right) \right)+2\cos\left( \frac{\mathrm{kL}}{2} \right)\exp\left( \mathrm{ik}\left( 2h+\frac{L}{2} \right) \right) \right)$ | (A4) |
|  | $+\left[ \mathrm{Ci}\left( u_{0}^{'} \right)-Ci\left( u_{4}^{'} \right)-iSi\left( u_{0}^{'} \right)-iSi\left( u_{4}^{'} \right) \right]\left( \exp\left( -ik\left( 2h \right) \right)+2\cos\left( \frac{\mathrm{kL}}{2} \right)\exp\left( -ik\left( 2h+\frac{L}{2} \right) \right) \right)$ |  |
|  | $+\left[ \mathrm{Ci}\left( u_{2} \right)-Ci\left( u_{4} \right)-iSi\left( u_{2} \right)+iSi\left( u_{4} \right) \right]\exp\left( \mathrm{ik}\left( 2h+L \right) \right)$ |  |
|  | $+\left[ \mathrm{Ci}\left( u_{2}^{'} \right)-Ci\left( u_{4}^{'} \right)-iSi\left( u_{2}^{'} \right)+iSi\left( u_{4}^{'} \right) \right]\exp\left( -ik\left( 2h+L \right) \right)$ |  |
|  | $+\left[ \mathrm{Ci}\left( u_{1} \right)-Ci\left( u_{3} \right)-iSi\left( u_{1} \right)+iSi\left( u_{3} \right) \right]\exp\left( \mathrm{ik}\left( 2h-L \right) \right)$ |  |
|  | $\left. \begin{matrix} \\ +[Ci(u_{1}^{'})-Ci(u_{3}^{'})-iSi(u_{1}^{'})-iSi(u_{3}^{'})]exp(-ik(2h-L))] \\ \end{matrix} \right].$ |  |

Where for perpendicular case $u_{0}=k\left( \sqrt{R^{2}+(2h)^{2}}+2h \right),u_{0}^{'}=k\left( \sqrt{R^{2}+(2h)^{2}}-2h \right),$

$$u_{1}=k\left( \sqrt{R^{2}+(2h-L)^{2}}+\left( 2h-L \right) \right),u_{1}^{'}=k\left( \sqrt{R^{2}+(2h-L)^{2}}-\left( 2h-L \right) \right),$$

$$u_{2}=k\left( \sqrt{R^{2}+(2h+L)^{2}}+\left( 2h+L \right) \right),u_{2}^{'}=k\left( \sqrt{R^{2}+(2h+L)^{2}}-\left( 2h+L \right) \right),$$

$$u_{3}=k(\sqrt{R^{2}+(2h-L/2)^{2}}+(2h-L/2)),u_{3}^{'}=k(\sqrt{R^{2}+(2h-L/2)^{2}}-(2h-L/2)),$$

$$u_{4}=k(\sqrt{R^{2}+(2h+L/2)^{2}}+(2h+L/2)),u_{4}^{'}=k(\sqrt{R^{2}+(2h+L/2)^{2}}-(2h+L/2)).$$

And the coefficients for the aligned case are: $u_{0}=k\left( \sqrt{R^{2}+(2h)^{2}}+R \right),u_{0}^{'}=k\left( \sqrt{R^{2}+(2h)^{2}}-R \right),$

$$u_{1}=k\left( \sqrt{\left( 2h \right)^{2}+(R-L)^{2}}+\left( R-L \right) \right),u_{1}^{'}=k\left( \sqrt{\left( 2h \right)^{2}+(R-L)^{2}}-\left( R-L \right) \right),$$

$$u_{2}=k\left( \sqrt{\left( 2h \right)^{2}+(R+L)^{2}}+\left( R+L \right) \right),u_{2}^{'}=k\left( \sqrt{{(2h)}^{2}+(R+L)^{2}}-\left( R+L \right) \right),$$

$$u_{3}=k(\sqrt{\left( 2h \right)^{2}+(R-L/2)^{2}}+(R-L/2)),u_{3}^{'}=k(\sqrt{\left( 2h \right)^{2}+(R-L/2)^{2}}-(R-L/2)),$$

$$u_{4}=k(\sqrt{\left( 2h \right)^{2}+(R+L/2)^{2}}+(R+L/2)),u_{4}^{'}=k(\sqrt{\left( 2h \right)^{2}+(R+L/2)^{2}}-(R+L/2)).$$

**S2. Energy transfer in free space between aligned dipoles**

**a**

**b**

**
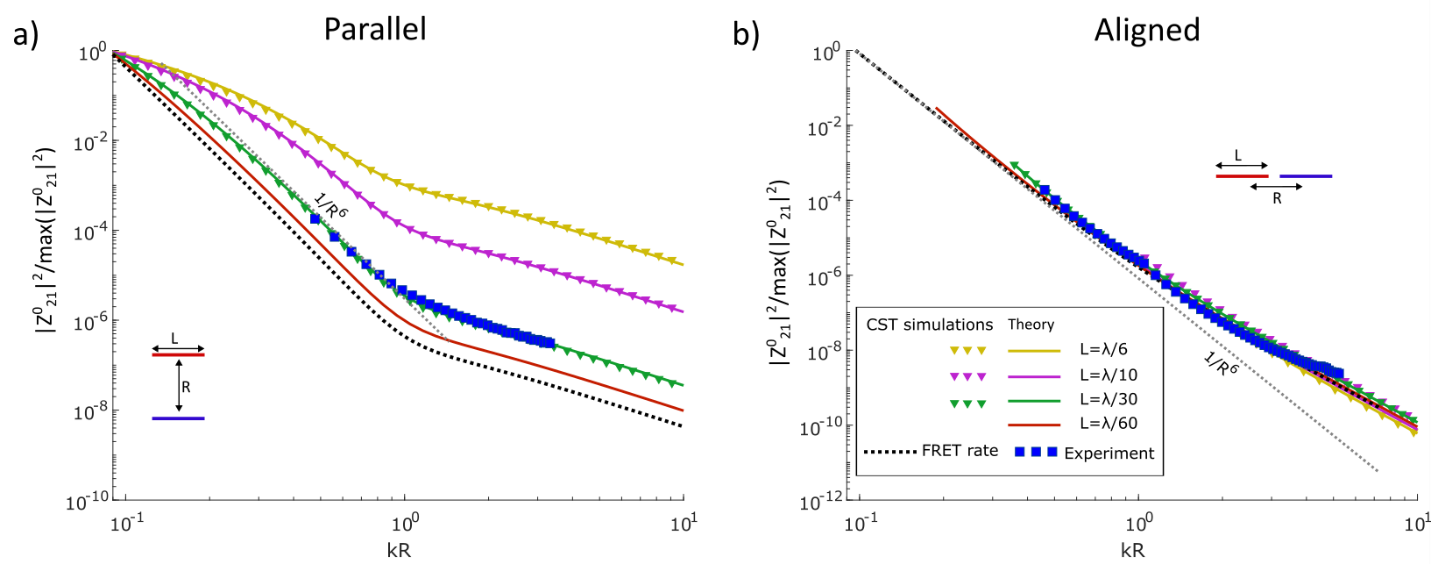
**
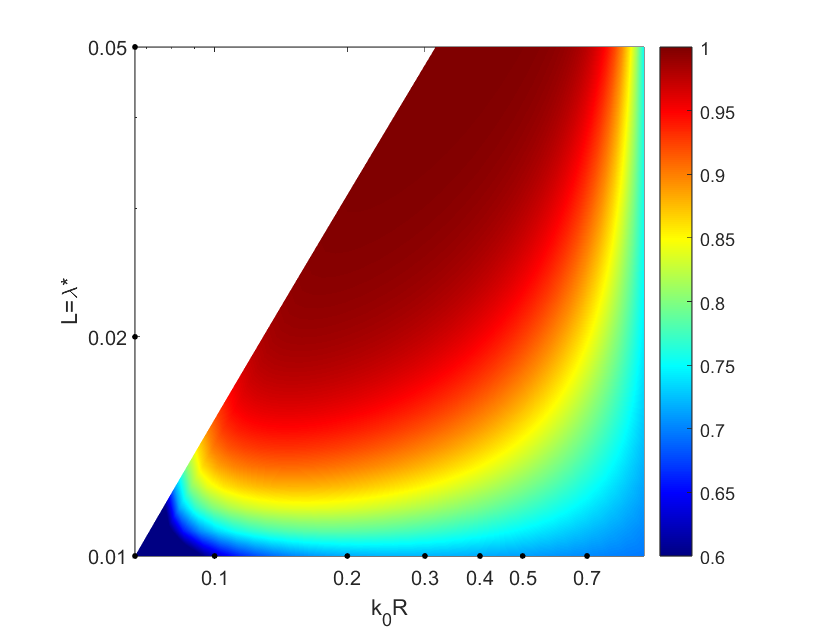


**Figure S1.** Dipole-dipole energy transfer in free space for the aligned configuration. (a) Calculated free space mutual impedance $\left| Z_{{21}_{align}}^{0} \right|^{2}$ normalized by its maximum for two parallel antennas with finite lengths L. The black dashed curve corresponds to the energy transfer between ideal point dipoles from Green’s function theory, which serves as a reference. Blue markers are experimental data recorded for L = λ/30. Triangular markers are numerical simulations using CST microwave studio. (b) Relative error (linear scale) of the normalized mutual impedance compared to point dipoles as a function of the dipole length and mutual separation.

**S3. FRET rate enhancement between point dipoles near a PEC mirror**


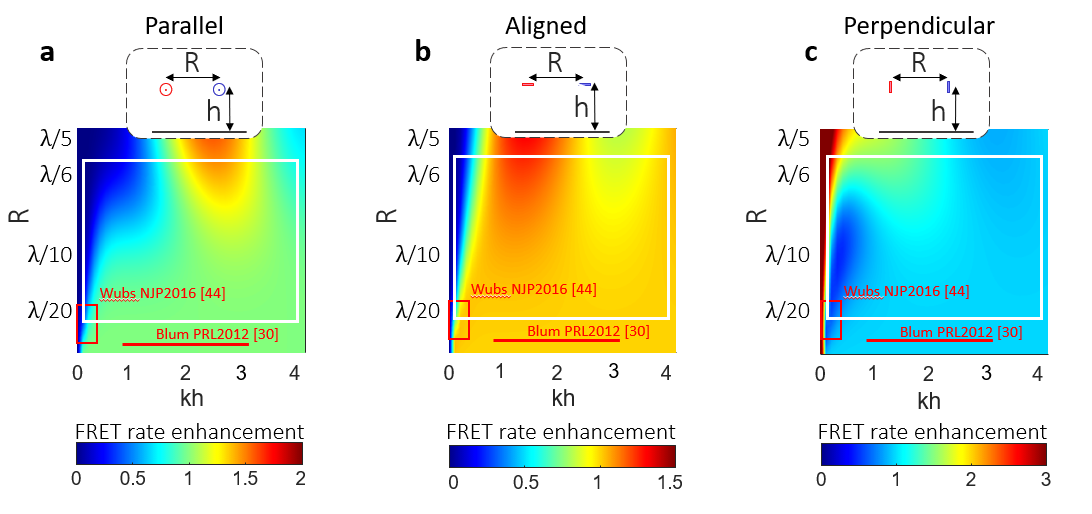


**Figure S2.** FRET rate enhancement between point-like dipoles near a PEC mirror calculated with Green’s function formalism for (a) parallel, (b) aligned, and (c) perpendicular orientations. $R$ is the dipole-dipole distance, $k$ is the wavenumber, and $h$ is the dipole’s distance from the mirror. Red boxes denote the relative ranges studied previously in optics and the white box denotes the experimental range considered here.

**S4. Energy transfer between aligned dipoles in presence of a PEC mirrror**

**
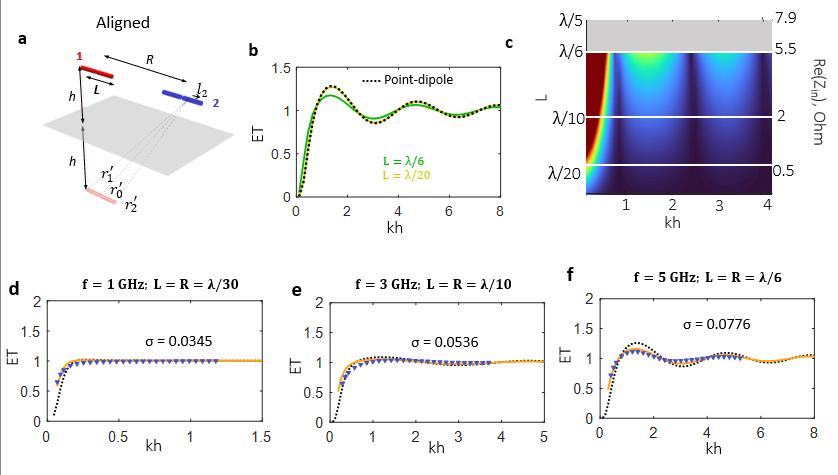
**

**Figure S3.** Energy transfer between two finite antennas near a PEC mirror for aligned orientation. (a) Schematics of the antennas. (b) Energy transfer enhancement (with respect to free space) as a function of the distance to the PEC mirror for ideal point dipoles (black dashed line, Green’s function theory) and dipoles of finite lengths L (color lines, our analytical model). The separation between antennas is $kR$ = 1 ($R$=10 mm, 5 GHz frequency). (c) Relative error in the energy transfer enhancement of finite antennas compared to point dipoles. The right axis indicates the real part of the antenna input impedance. The grey area in corresponds to the physically prohibited region due to finite dipole lengths. (d-f) Experimental validation of the antenna length influence on the energy transfer enhancement near a PEC mirror. Blue triangles are experimental results, orange curves are the result of our analytical model and the dashed black curves are predictions from Green’s function theory. The root mean square errors $\sigma$ between the experimental results and the Green’s theory predictions are shown in each graph.
